# Small Non-coding RNAs: New Class of Biomarkers and Potential Therapeutic Targets in Neurodegenerative Disease

**DOI:** 10.3389/fgene.2019.00364

**Published:** 2019-04-26

**Authors:** Callum N. Watson, Antonio Belli, Valentina Di Pietro

**Affiliations:** ^1^Neuroscience and Ophthalmology Research Group, Institute of Inflammation and Ageing, University of Birmingham, Birmingham, United Kingdom; ^2^National Institute for Health Research Surgical Reconstruction and Microbiology Research Centre, Queen Elizabeth Hospital Birmingham, Birmingham, United Kingdom; ^3^Beckman Institute for Advanced Science and Technology, University of Illinois at Urbana–Champaign, Urbana, IL, United States

**Keywords:** small non-coding RNAs, microRNAs, neurodegenerative disease, biomarkers, new therapeutic targets

## Abstract

Neurodegenerative diseases (NDs) are becoming increasingly prevalent in the world, with an aging population. In the last few decades, due to the devastating nature of these diseases, the research of biomarkers has become crucial to enable adequate treatments and to monitor the progress of disease. Currently, gene mutations, CSF and blood protein markers together with the neuroimaging techniques are the most used diagnostic approaches. However, despite the efforts in the research, conflicting data still exist, highlighting the need to explore new classes of biomarkers, particularly at early stages. Small non-coding RNAs (MicroRNA, Small nuclear RNA, Small nucleolar RNA, tRNA derived small RNA and Piwi-interacting RNA) can be considered a “relatively” new class of molecule that have already proved to be differentially regulated in many NDs, hence they represent a new potential class of biomarkers to be explored. In addition, understanding their involvement in disease development could depict the underlying pathogenesis of particular NDs, so novel treatment methods that act earlier in disease progression can be developed. This review aims to describe the involvement of small non-coding RNAs as biomarkers of NDs and their potential role in future clinical applications.

## Introduction

Neurodegenerative diseases (NDs) are classified as a class of disorders affecting the central nervous system and they are characterized by the progressive loss of neuronal tissues. NDs are age-dependent disorders which are increasing internationally, due to the ever increasing elderly population, which is leaving greater numbers of people subjected to the chronic, debilitating nature of these incurable diseases (Heemels, 2016). Currently, the most represented NDs are: Alzheimer’disease (AD) with 5 million people affected in America only, followed by Parkinson’s diseases (PD) with 1 million people; multiple sclerosis (MS) 400,000; Amyotrophoic lateral sclerosis (ALS) 30,000 and Huntington’s disease (HD) with 3,000 incidents (Agrawal and Biswas, 2015).

Some treatments for ND have aimed to reduce the syndrome of NDs; these include L-dopa and deep brain stimulation in PD (Groiss et al., 2009; Nagatsua and Sawadab, 2009). However, very few have aimed to slow or reverse ND development, and those that have been investigated e.g., stem cell therapy (Chung et al., 2002; Rachakonda et al., 2004) highlight the requirement for more research. Late diagnosis leads to strategic treatment being ineffective due to irreversible disease progression (Sheinerman and Umansky, 2013). This has been reported for example, on anti-AD therapies in late-stage clinical trials (including dimebon of Medivation and Pfizer, solanezumab of Eli Lilly and bapineuzumab of Pfizer and Johnson & Johnson). Biomarkers for early diagnosis could prevent or limit disease development through prophylactic or early treatment, which has ignited interest. Currently, the most accurate diagnosis relies on neuropathology, mainly based on autopsy, or in the measurement of cerebrospinal fluid (CSF) proteins, such as tau or Aβ- in AD, which requires invasive procedures. However, blood proteins, such as Aβ1-42 peptide in AD or cytokines for ALS or HD (Agrawal and Biswas, 2015), as well as genetics diagnostics markers such as ApoE isoforms in AD or α-synuclein or Parkin for PD, have also demonstrated potential clinical utility (Agrawal and Biswas, 2015).

Neuroimaging techniques can also help to make the correct diagnosis and monitor the progress of NDs. Magnetic resonance imaging (MRI) is one of the most widely used neuroimaging techniques used for AD (Jack et al., 2011; McKhann et al., 2011) and for dementia with Lewy bodies (DLB) (Ciurleo et al., 2014). Magnetic resonance spectroscopy (MRS) has also showed promise in early diagnosis of PD and traumatic brain injury, measuring metabolic dysfunctions and irreversible neuronal damage (Vagnozzi et al., 2008).

Recently, a new class of circulating RNAs – non-coding RNAs – have been re-evaluated and are being considered as potential biomarkers. After years of the belief that 98% of the genome was “junk” due to its non-coding nature it was realized these genes had biologically functionality. Non-coding genes include introns, pseudogenes, repeat sequences and *cis*/*trans-*regulatory elements that function as RNA without translation. Estimations have suggested that 99% of total RNA content is made up of non-coding RNA, with numbers of validated non-coding RNAs (ncRNAs) increasing every year (Palazzo and Lee, 2015).

Currently ncRNAs can be defined by length – small 18–200 nts and long >200nts – or functionality with housekeeping ncRNAs such as ribosomal RNAs (rRNAs) and transfer RNAs (tRNAs) or regulatory ncRNAs like microRNAs (miRNAs), small nuclear RNAs (snRNAs), piwi-interacting RNA (piRNAs), tRNA derived small RNAs (tsRNAs) and long non-coding RNAs (lncRNAs) (Dozmorov et al., 2013). Nonetheless, difficulty distinguishing categories persists due to the crossover of properties.

Small non-coding RNAs (sncRNAs) have diverse roles, which in conjunction with other molecules involve gene regulation through either RNA interference, RNA modification or spliceosomal involvement (Table 1). Consequently, during disease progression their expression can alter. MiRNAs are the most studied sncRNA as biomarkers with involvement in various diseases including cancers, aging and neurodegenerative disease (Calin and Croce, 2006; Grasso et al., 2014; Di Pietro et al., 2017). Other sncRNAs have shown promise as biomarkers, with links to neurodegenerative disease (Munoz-Culla et al., 2016). There is the potential for multiple sncRNA biomarkers for neurodegenerative diseases, which if found, could aid diagnosis in a clinical setting while demonstrating the processes underpinning the disease development. In future, this could produce novel therapies to treat neurodegenerative diseases using original methodologies.

**Table 1:** Classification of types of small non-coding RNAs.

Type of small non-coding RNA	Size (nts)	Function
MicroRNA (miRNA)	∼22	Ago – RNAi
Small nuclear RNA (snRNA)	∼150	Spliceosome components
Small nucleolar RNA (snoRNA)	60–140	RNA modification
Piwi-interacting RNA (piRNA)	26–31	PIWI – RNAi
tRNA derived small RNA (tsRNA)	15–50	Diverse


In this review, we consider the evolving role of sncRNAs and discuss their involvement in neurodegenerative disease with particular emphasis on their potential as biomarkers.

## MicroRNA

**FIGURE 1:**
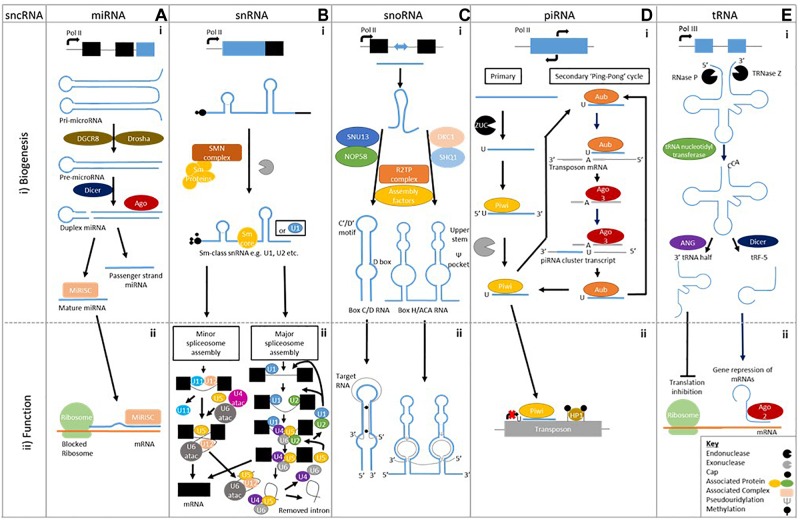
Biogenesis of sncRNAs and an example of their biological function. **A (i)** MicroRNAs are single stranded ∼22 bp sequences formed from double stranded precursors **(ii)** that prevent mRNA translation. **B (i)** Small nuclear RNAs biogenesis is made up of two classes Sm class snRNA and Lsm-class snRNA (Not shown), **(ii)** which form the major and minor spliceosome. **C (i)** Small nucleolar RNAs have two different classes formed using different machinery; Box C/D RNA and Box H/ACA RNA, **(ii)** which cause methylation and pseudouridylation respectively. **D (i)** Piwi interacting RNAs are formed by either primary alone or by both primary and secondary biogenesis **(ii)** that prevent transposon translation through methylation. **E (i)** Transfer RNA cleavage forms transfer RNA derived fragments to be formed, **(ii)** which can prevent translation or cause gene repression.

MiRNAs are the most studied sncRNA. Their biogenesis commences with the formation of a pri-miRNA made up of two stem-loop structure. A Drosha and DGCR8 complex cleaves the pri-miRNA to form a single stem-loop pre-miRNA. Dicer cleaves the pre-miRNA to create a double stranded miRNA, which is loaded onto Argonaute family of proteins to form the miRISC complex (Figure 1Ai). Accompanied to the miRISC complex, miRNAs regulate gene expression post-transcriptionally through degradation and repression of mRNA sequences by an Argonaute family protein mediated method (Figure 1Aii; O’Brien et al., 2018). A single miRNA can have multiple targets, likewise a target mRNA can be bound to by many different miRNAs, to enable more diverse signaling patterns.

MiRNAs show specific signaling in the brain, and were also found differentially expressed in bio-fluids. Although there is no consistent consensus on particular miRNAs or brain area yet, and no specific miRNA overlap between brain tissues and bio-fluids (as reported in Table 2) these findings certainly provide insights in the study of NDs pathogenesis.

**Table 2:** MiRNAs with an involvement in the neurodegenerative disease development.

Neurodegenerative disease	Source	miRNA
Alzheimer’s disease	Whole blood	miR-106b-5p, miR-106a-5p, miR-107, miR-9-5p (Yilmaz et al., 2016a) Let-7d-5p, Let-7f-5p, miR-107, miR-26a-5p, miR-26b-5p (Leidinger et al., 2013) miR-142-5p (Sorensen et al., 2016)
	Brain tissues	miR-9, miR-128 (Lukiw, 2007) miR-26a, miR-27b, miR-30e-5p, miR-34a, miR-92, miR-125, miR-145, miR-200c, miR-381, miR-422a, miR-423 miR-9, miR-132, miR-146b, miR-212 (Cogswell et al., 2008) miR-197, miR-511, miR-320, let-7i, miR-101, miR-106b, miR-15a, miR-181c, miR-19b, miR-22, miR-210, miR-26b, miR-29b-1, miR-363, miR-9, miR-93 (Hebert et al., 2008) miR-29a (Shioya et al., 2010) miR-26b (Absalon et al., 2013) miR-370, miR-328, miR-138, miR-132 and miR-15a (Bekris et al., 2013)
	CSF	let-7f, miR-105, miR-125a, miR-135a, miR-138, miR-141, miR-151, miR-186, miR-191, miR-197, miR-204, miR-205, miR-216, miR-302b, miR-30a5p, miR-30a3p, miR-30b, miR-30c, miR-30d, miR-32, miR-345, miR-362, miR-371, miR-374, miR-375, miR-380-3p, miR-429, miR-448, miR-449, miR-494, miR-501, miR-517, miR-517b, miR-518b, miR-518f, miR-520a^∗^, miR-526a, miR-10a, miR-10b, miR-125, miR-126^∗^, miR-127, miR-142-5p, miR-143, miR-146b, miR-154, miR-15b, miR-181a, miR-181c, miR-194, miR-195, miR-199a^∗^, miR-214, miR-221, miR-328b, miR-422, miR-451, miR-455, miR-497, miR-99a (Bekris et al., 2013) miR-9, miR-125b, miR-146a, miR-155 (Alexandrov et al., 2012) let-7b (Lehmann et al., 2012) miR-146a, miR-155 (Lukiw et al., 2012) miR27a-3p (Sala Frigerio et al., 2013) miR-100, miR-146a, miR-296, miR-3622b-3p, miR-4467, miR-505, miR-766, miR-103, miR1274a, miR-375, miR-708, miR-219 (Denk et al., 2015)
	Plasma	miR-142-3p, miR-483-5p (Nagaraj et al., 2017) miR-125b-5p, miR-29b-3p, miR-3065-5p, miR-342-3p/5p (Lugli et al., 2015) miR-107 (Wang et al., 2008) miR-34a (Bhatnagar et al., 2014) miR-146a, miR-34a (Kiko et al., 2014) Let-7d-5p, Let-7g-5p, miR-142-3p, miR-15b-5p, miR-191-5p (Kumar et al., 2013)
	Serum	miR-143, miR-146a, miR-93 (Dong et al., 2015) miR-125b, miR-26b (Galimberti et al., 2014) Let-7d-5p, Let-7g-5p, miR-191-5p, miR-26b-3p, miR-30e-5p, miR-342-3p, miR-483-3p (Tan et al., 2014b) miR-125b, miR-181c, miR-9 (Tan et al., 2014a) miR-106b-3p, miR-181c-3p, miR-26a-5p (Guo R. et al., 2017) Let-7f-5p, miR-26b-5p, miR-501-3p (Hara et al., 2017) miR-125b (Jia and Liu, 2016) miR-106a-5p, miR-106b-3p, miR-143-3p, miR-15b-3p, miR-3065-5p, miR-30e-5p, miR-342-3p, miR-93-5p (Cheng et al., 2015) miR-181c (Geekiyanage et al., 2012) miR-455-3p (Kumar et al., 2017) miR-222 (Zeng et al., 2017) miR-29c-3p, miR-19b-3p (Wu et al., 2017)
	PBMCs	miR-29b (Villa et al., 2013) Let-7f, miR-34a (Schipper et al., 2007)
Early onset Parkinsons disease (EOPD)	Whole blood	miR-1, miR-22, miR-29a (Margis et al., 2011)
	Brain tissues	miR-34b,c (Minones-Moyano et al., 2011)
	Plasma	miR-331-5p (Cardo et al., 2013)
	Serum	miR-141, miR-146b-5p, miR-193a-3p, miR-214 (Dong et al., 2016)
Late onset Parkinson’s disease (LOPD)	Whole Blood	miR-103a, miR29a, miR-30b (Serafin et al., 2015) miR-3143, miR-335-3p, miR-4671-3p, miR-561-3p, miR-579-3p (Yilmaz et al., 2016b)
	Brain tissues	miR-34b,c (Minones-Moyano et al., 2011) miR-181a,b,c,d, miR-22, miR-29a,b,c (Liao et al., 2013) miR-106a, miR-21, miR-224, miR-26b, miR-301b, miR-373 (Alvarez-Erviti et al., 2013) miR-205 (Cho et al., 2013) miR-135b, miR-198, miR-485-5p, miR-548d (Cardo et al., 2014) Let-7i-3p/5p, miR-10b-5p, miR-1224, miR-127-3p, miR-127-5p, miR-16-5p, miR-184, miR-29a-3p (Hoss et al., 2016) miR-144, miR-145, miR-199b, miR-221, miR-488, miR-543, miR-544, miR-7 (Tatura et al., 2016) miR-225, miR-236, miR-46 (Wake et al., 2016)
	CSF	Let-7g-3p, miR-1224-5p, miR-127-3p, miR-128, miR-132-5p, miR-19a,b, miR-212-3p, miR-370, miR-409-3p, miR-4448, miR-485-5p, miR-873-3p (Burgos et al., 2014) Let-7g-3p, miR-1, miR-103a, miR-10a-5p, miR-119a, miR-126, miR-127-3p, miR-132-5p, miR-136-3p, miR-151, miR-153, miR-16-2, miR-19b-3p, miR-22, miR-26a, miR-28, miR-29a,c, miR-301a, miR-30b, miR-331-5p, miR-370, miR-374, miR-409-3p, miR-433, miR-485-5p, miR-873-3p (Gui et al., 2015) miR-1249, miR-1274b, miR-150, miR-16, miR-18b, miR-199b, miR-20a, miR-21, miR-320a,b, miR-378c, miR-4293, miR-671, miR-769, miR-92b (Soreq et al., 2013)
	Plasma	miR-222, miR-505, miR-626 (Khoo et al., 2012)
	Serum	miR-19b, miR-29a,c (Botta-Orfila et al., 2014) miR-133b (Zhao et al., 2014) miR-29a,b,c (Bai et al., 2017) miR-146a, miR-214, miR-221, miR-29c (Ma et al., 2016) miR-1294, miR-16-2-3p, miR-30a,e, miR-338-3p (Burgos et al., 2014) miR-148b, miR-223, miR-24, miR-30c, miR-324-3p (Vallelunga et al., 2014) miR-15b, miR-181a, miR-185, miR-195, miR-221 (Ding et al., 2016)
	PBMCs	miR-126, miR-126^∗^, miR-147, miR-151-3p,5p, miR-199a-3p,5p, miR-199b, miR-19b, miR-26a, miR-28-5p, miR-29b,c, miR-301a, miR-30b,c, miR-335, miR-374a,b (Martins et al., 2011) miR-155, miRNA-146a (Caggiu et al., 2018)
Amyotrophic lateral sclerosis (ALS)	Whole Blood	let-7a-5p, let-7d-5p, let-7f-5p, let-7g-5p, let-7i-5p, miR-103a-3p, miR-106b-3p, miR-128-3p, miR-130a-3p, miR-130b-3p, miR-144-5p, miR-148a-3p, miR-148b-3p, miR-15a-5p, miR-15b-5p, miR-151a-5p, miR-151b, miR-16-5p, miR-182-5p, miR-183-5p, miR-186-5p, miR-22-3p, miR-221-3p, miR-223-3p, miR-23a-3p, miR-26a-5p, miR-26b-5p, miR-27b-3p, miR-28-3p, miR-30b-5p, miR-30c-5p, miR-342-3p, miR-425-5p, miR-451a, miR-532-5p, miR-550a-3p, miR-584-5p, miR-93-5p (Liguori et al., 2018)
	CSF	miR-150, miR-99b, miR-146a, miR-27b, miR-328, miR-532-3p (Butovsky et al., 2012) miR-132-5p, miR-132-3p, miR-143-3p, miR-143-5p, miR-574-5p (Freischmidt et al., 2013) miR-338-3p (De Felice et al., 2014) miR-181a-5p, miR-21-5p, miR-195-5p, miR-148-3p, miR-15b-5p, miR-let7a-5p, miR-let7b-5p, miR-let7f-5p (Benigni et al., 2016) miR-124-3p, miR-127-3p, miR-143-3p, miR-125b-2-3p, miR-9-5p, miR-27b-3p, miR-486-5p, miR-let7f-5p, miR-16-5p, miR-28-3p, miR-146a-3p, miR-150-5p, miR-378a-3p, miR-142-5p, miR-92a-5p (Waller et al., 2017b)
	Plasma	miR-4649-5p, miR-4299 (Takahashi et al., 2015) miR-424, miR-206 (de Andrade et al., 2016) miR-206, Pairs miR-206/miR-338-3p, miR-9^∗^/miR-129-3p, miR-335-5p/miR-338-3p (Sheinerman et al., 2017)
	Serum	miR-132-3p, miR-132-5p, miR-143-3p, miR-143-5p, let-7b (Freischmidt et al., 2013) miR-206, miR-106b (Toivonen et al., 2014) miR-4745-5p, miR-3665, miR-4530, miR-1915-3p (Freischmidt et al., 2014) miR-1825, miR-1234-3p (Freischmidt et al., 2015) miR-206, miR-133a, miR-133b, miR-146a, miR-149^∗^, miR-27a (Tasca et al., 2016) miR-1, miR-133a-3p, miR-133b, miR-144-5p, miR-192-3p, miR-195-5p, miR-19a-3p, let-7d-3p, miR-320a, miR-320b, miR-320c, miR-425-5p, miR-139-5p (Raheja et al., 2018) miR-206, miR-143-3p, miR-374b-5p (Waller et al., 2017a) miR-142-3p, miR-1249-3p (Matamala et al., 2018)
Huntington’s disease	Brain tissues	miR-9/miR-9^∗^, miR-124a, miR-132 (Packer et al., 2008) miR-10b-5p, miR-196a-5p, miR-615-3p, miR-10b-3p, miR-1298-3p, miR-196b-5p, miR-302a-3p, miR-1247-5p, miR-144-3p, miR-223-3p, miR-3200-3p, miR-302a-5p, miR-1264, miR-6734-5p, miR-144-5p, miR-138-2-5p, miR-431-5p, miR-132-3p, miR-200c-3p, miR-23b-5p, miR-448, miR-486-3p, miR-490-5p, miR-5695, miR-885-5p, miR-1224-5p, miR-1298-5p, miR-142-3p, miR-346, miR-891a-5p, miR-16-2-3p, miR-363-3p, miR-148a-3p, miR-199a-5p, miR-4449, miR-106a-5p, miR-142-5p, miR-549a, miR-214-5p, miR-141-3p, miR-5680, miR-3065-5p, miR-224-5p, miR-4787-3p, miR-452-5p, miR-129-1-3p, miR-4443, miR-101-5p, miR-483-5p, miR-2114-5p, miR-1185-1-3p, miR-670-3p, miR-129-5p, miR-135b-5p, miR-194-5p, miR-208b-3p, miR-4488, miR-888-5p, miR-126-5p, miR-34c-5p, miR-218-1-3p, miR-150-5p, miR-486-5p, miR-433-3p, miR-219b-3p, miR-548n, miR-663b, miR-148a-5p, miR-29a-3p, miR-320b, miR-181a-3p, miR-153-5p, miR-28-5p, miR-7-2-3p, miR-877-5p, miR-3687, miR-4516, miR-3139, miR-663a, miR-34b-3p, miR-1538 (Hoss et al., 2015a)
	CSF	miR-520f-3p, miR-135b-3p, miR-4317, miR-3928-5p, miR-8082, miR-140-5p (Reed et al., 2018)
	Plasma	miR-10b-5p, miR-486-5p (Hoss et al., 2015b) miR-34b (Gaughwin et al., 2011) miR-877-5p, miR-223-3p/5p, miR-30d-5p, miR-128, miR-22-5p, miR-222-3p, miR-338-3p, miR-130b-3p, miR-425-5p, miR-628-3p, miR-361-5p, miR-942 (Diez-Planelles et al., 2016)


*^∗^Passenger miRNA strand.*

MiRNAs are best studied in Alzheimer’s disease (AD), which manifests itself as deposition of neurofibrillary tangles (NFT) and extracellular amyloid-β (Aβ), before neuronal degeneration and clinical symptoms materialize in the form of behavioral changes such as memory issues. NFT, Aβ and neuronal degeneration have been associated with dysregulation of miRNA gene expression, which could emanate from altered Aβ or Tau metabolism. MiRNAs effect Aβ metabolism by interacting with amyloid precursor protein (APP) through direct binding of the 3′untranslated region (3′UTR) to the APP mRNA, indirect inhibition through downregulation of Beta-secretase 1 (BACE1) and ATP-binding cassette transporter (ABCA1) or regulating alternative APP splicing. MiRNAs also affect Tau through regulation of microtubule associated protein tau (MAPT) splicing, affecting tau isoforms 3R and 4R. Direct or indirect binding either modulates phosphorylated Tau-associated protein kinases or influences degradation of phosphorylated tau by binding 3′-UTR BCL2 associated athanogene 2 (BAG2) mRNA (Zhao et al., 2017).

MiRNAs have an established involvement in neurobiological functions and pathogenesis of numerous other neurodegenerative diseases (Serafin et al., 2014; Fransquet and Ryan, 2018; Ricci et al., 2018). Mitochondrial dysfunction caused by miRNA dysregulation leads to oxidative stress, which causes cell death, α-synuclein aggregation and neurodegeneration known to be present in PD (Spano et al., 2015). In ALS, both TAR DNA binding protein (TARDBP) and fused in sarcoma (FUS) are well-established causative genes, which are involved in miRNA processing. TARDBP has specific roles in facilitation of post-transcriptional processing achieved through association directly with miRNA or processing factors such as Dicer (Kawahara and Mieda-Sato, 2012). FUS regulates miRNA-mediated gene silencing through facilitation of the interaction between miRNA, mRNA and RISC components (Zhang et al., 2018). In HD, a miRNA formulation is being trailed as therapeutic agents to alter the aberrant Huntingtin (HTT) protein expression (Aronin and DiFiglia, 2014).

MiRNA involvement in ND development has demonstrated the capability of distinguishing between disease subtypes and shown promise for future stratification. For example in AD, 30 differentially regulated miRNAs found in the brain and blood of AD patients were assigned to different Braak stages, a methodology for classifying AD pathology, with 10 associated with Braak stage III (hsa-mir-107, hsa-mir-26b, hsa-mir-30e, hsa-mir-34a, hsa-mir-485, hsa-mir200c, hsa-mir-210, hsa-mir-146a, hsa-mir-34c, and hsa-mir-125b) (Swarbrick et al., 2019). Likewise in PD, miR-331-5p is differentially expressed in plasma of early onset Parkinson’s disease (EOPD) patients, which was not seen in late onset Parkinson’s disease (LOPD) patients (Cardo et al., 2013; Table 2). Studies comparing between subtypes of NDs are still in the minority and more are required to understand the true capability of miRNA markers in stratification of NDs.

## Small Nuclear RNAs

Small nuclear RNAs (snRNAs), the component parts of the spliceosome – responsible for removal of non-coding introns from precursor mRNA – are highly conserved uridine rich sequences with five snRNAs making up its spine; U1, U2, U4, U5, and U6. These snRNAs combine with partner proteins to form the small nuclear ribonucleoprotein (snRNPs) complex, which is essential pre-mRNA splicing to enable production of functional mRNA for protein translation.

Sm-class snRNAs are synthesized by RNA polymerase II and after transcription contain a 7-methylguanosine cap, Sm-protein binding site and 3′ stem-loop. The latter two are recognized by the SMN complex, which recruits a set of Sm proteins to create the Sm-core RNP. Following this, the cap undergoes hypermethylation by trimethylguanosine synthase-1 (TSG1) creating a 2,2,7-trimethylguanosine cap. The 3′ end is then trimmed by an unknown exonuclease before subsequent maturation through modifications (Matera et al., 2007; Figure 1Bi).

Two types of spliceosome “major” and “minor” (0.35% of all introns) can be assembled. Major spliceosome assembly commences by U1 interacting with the 5′ splice site while U2 snRNP binds to the branch point sequence. This leads to the recruitment of the premade U4/U6.U5 tri-snRNP complex, in this state the spliceosome is inactive. After destabilization or release of either U1 or U4, the spliceosome becomes active. The active spliceosome undergoes two phases of catalysis leading to its dissociation – including U2, U5, and U6 that are recycled – when it releases the mRNA, as mRNP (Wahl et al., 2009; Figure 1Bii). The minor spliceosome has divergent and highly conserved 5′ splice site and branch point sequences, which interact with U5 as well as alternative factors U11, U12, and U4atac/U6atac that are functional analog of its major counterpart (Verma et al., 2018; Figure 1Bii). Both spliceosomes show the capability to contribute to the development of neurodegenerative disease, demonstrating snRNA involvement (Bai et al., 2013; Tsuiji et al., 2013; Ratti and Buratti, 2016; Jutzi et al., 2018).

In sporadic and familial AD, U1 snRNP subunits – including U1-70K and U1A – were present in cytoplasmic aggregates, which occurs by the basic-acidic dipeptide (BAD) domain binding to tau in U1-70K (Bishof et al., 2018). Inordinate levels of unspliced RNA also reside, caused by dysregulation of RNA processing. In conjunction with evidence that inhibition of U1 snRNP increases APP, this implicates U1 snRNP dysregulation in the pathogenesis of AD (Bai et al., 2013; Hales et al., 2014a,b). Recent evidence has shown abnormal expression of U1 snRNA can cause premature cleavage of pre-mRNA via polyadenylation (PCPA) at the 3′ poly-A site. This affects splicing and could demonstrate a novel AD causing pathology (Cheng et al., 2017) (Table 3).

**Table 3:** Interactions of small non-coding RNAs with Neurodegenerative diseases.

sncRNA	Disease	Interaction
snRNA major spliceosome	AD	U1 snRNPs present in cytoplasmic aggregates
	SMA	SMN1 gene dysregulation alters U snRNA levels
	Neurodegeneration	U2snRNA mutation alters pre-mRNA splicing
	ALS FTD	A disease related di-peptide repeat C90RF72 interacts with U2 snRNP
	RP	Mutation found in PRPF4 which encodes U4/U6 di-snRNP protein
snRNA minor spliceosome	ALS	Decreased U12 snRNA in spinal motor neurones
		Decreased TDP-43 disrupts U12 mediated pre-mRNA splicing
		FUS mutants cannot bind U11 so decreased minor intron splicing
snoRNA	AD	Differential expression of two C/D box snoRNAs e307 and e470 in mouse model
	ASD	SNORD115 duplication in mouse causes abnormal brain development
piRNA	AD	9 piRNAs found to be differentially regulated in AD risk variant patients (6 APOE and 3 RNU6-560P)
	PD	70 differentially expressed piRNAs in combined patient tissue and cells
tsRNA	ALS	ANG mutants implicated in pathogenesis
	PD	A subset of ALS-associated ANG mutants
	Intellectual disability	NSun2 mutation causes 5′tiRNA accumulation
	PCH	CLP1 gene mutation disruption of tRNA splicing
	Neurodegenerative patient	KAE1 gene mutation alters tRNA modification


U snRNAs are also associated with spinal muscular atrophy (SMA). SMN1 gene dysregulation alters U snRNA levels through its role in U snRNA biosynthesis; nonetheless, the underlying pathology is still unclear (Zhang et al., 2013). Many studies have proposed a reduction in U snRNAs is key to SMA pathology due to their involvement in mRNA processing, with U1 and U11 of particular interest (Gabanella et al., 2007; Zhang et al., 2008). In contrast, U snRNAs can accumulate in the motor neurons of ALS patient spinal cords when compared to control patients, to cause defects showing that U snRNA level can depict disease state, depending of cell type (Tsuiji et al., 2013).

More recently, when considering induced pluripotent stem cell (iPSC) derived motor neurones cultures, a study suggested that an imbalanced ratio of variant U1 to U1 might cause the SMA phenotype rather than an overall reduction in U1 snRNA (Vazquez-Arango et al., 2016). Demonstrating that purely measuring U snRNA level may be an oversimplified measurement and variant U snRNA could indicate the underlying pathophysiology of aberrant spliceosome related neurodegeneration.

Other U snRNAs studied in neurodegenerative disease include U2. A U2 snRNA mutation causes neuron degeneration, through altering pre-mRNA splicing at select splice sites that are associated with alternative pre-mRNA splicing (Jia et al., 2012). In addition, a dipeptide repeat (C90RF72) linked to both ALS and frontotemporal dementia (FTD), interacts and interferes with U2 snRNP. In patient derived cells, this led to mislocalisation but mis-splicing linked to ALS/FTD has yet to be established (Yin et al., 2017).

Mutations found within the gene PRPF4 – which encodes hPrp4 a U4/U6 di-snRNP protein – undertake an important role in the development of retinitis pigmentosa (RP) (Chen et al., 2014). hPrp4 is known to interact with CypH and hPrp3 to regulate the stability of the tri-snRNP, U4/U6.U5. Thus, aberrant splicing could cause RP through direct or indirect mechanisms that have been hypothesized, but not defined.

The minor spliceosome has ND relevance as in ALS, TDP-43 functionality decreases (Colombrita et al., 2012), which reduces the number Gemini of coiled bodies (GEMs). GEMs contribute to U12 snRNA biogenesis, so in spinal motor neurones of ALS patients there was a decrease of U12 snRNA and U11/U12 snRNP, which may disrupts pre-mRNA splicing (Ishihara et al., 2013). Additionally, an ALS mutant (P525L) cannot promote minor intron splicing due to an aberrant FUS gene that routinely binds to U11 snRNP to direct splicing. This leads to mislocalisation of FUS-trapped U11 and U12 snRNAs, which form aggregates in the cytoplasm so incorrect splicing results (Reber et al., 2016). In addition, a cerebral ataxia mutation RNU12 causes minor intron retention in homozygous mutant patients (Elsaid et al., 2017). When combined this demonstrates a likely role for minor intron splicing in motor neurone maintenance.

## Small Nucleolar RNAs

Small nucleolar RNAs (SnoRNAs) modify RNA through there conserved motifs, with boxes C/D guiding methylation and H/ACA guiding pseudouridylation, respectively (Ohtani, 2017; Figure 1Cii). Each class of snoRNAs displays a unique secondary structure composed of conserved proteins to form the defined C/D and H/ACA snoRNPs. SnoRNAs mainly target rRNA to modify functionally important regions of the ribosome (Decatur and Fournier, 2002) but other purposes include pre-rRNA endonucleolytic processing (Tollervey and Hurt, 1990), guiding snRNAs such as U6 snRNA (Tycowski et al., 1998) and more recently mRNA guiding (Sharma et al., 2016) or regulation of alternative splicing in pre-mRNAs (Falaleeva et al., 2016).

Box C/D snoRNP biogenesis commences when a protein complex of SNU13 and NOP58 is pre-formed and loaded onto the snoRNA with the help of HSP90/R2TP. This recruits assembly factors and the pre-snoRNPs are transferred to the Cajal bodies where final processing occurs. Box H/ACA RNPs biogenesis starts by SHQ1 and DKC1 combining to prevent to non-specific RNAs binding. SHQ1 is released with the help of the R2TP complex allowing DKC1 to bind H/ACA RNAs at the site of transcription. Numerous assembly factors including NHP2, NOP10, and NAF1 are present during this pre-snoRNP form. When NAF1 – which binds the C-terminal domain of RNA polymerase II to keep H/ACA RNP inactive – is replaced by GAR1, mature and functional H/ACA RNPs are produced. Both forms are transported to the nucleolus to elicit their actions (Massenet et al., 2017; Figure 1Ci).

A study showed differential regulation of two C/D box snoRNAs (e307 and e470) prior to the development of AD in mouse model. After formation of a β-amyloid plaque, this differential expression is no longer present, demonstrating that they could be useful in early diagnosis. No clear evidence of pathogenesis just hypothesized using bioinformatics methods (Gstir et al., 2014) (Table 3).

Despite the fact that autism spectrum disorder (ASD) might not be considered a neurodegenerative disease. Studies have found links in ASD with numerous snoRNA genes found to be differentially expressed using RNA-seq (Wright et al., 2017). Duplication of SNORD115 in mouse chromosome 7 that mirrors human chromosome 15q11-13 – duplication of this is one of the most common chromosomal abnormalities in ASD – has been shown to increase SNORD115 levels and results in abnormal brain development. In addition, SNORD115 (HBII-48 and HBII-52) levels are dysregulated in superior temporal gyrus of human ASD brain samples, which could explain 5-HT changes (Gabriele et al., 2014) and alternative splicing seen in ASD (Voineagu et al., 2011) as HBII-52 may regulate 5-HT2C receptor mRNA levels (Stamova et al., 2015) as well as alternative splicing (Kishore et al., 2010).

Another study demonstrated that maternal alcohol consumption in pregnancy alters the C/D box RNA levels in brain cells during abnormal fetal development. DNA methylation, microRNA and snoRNA levels altered with emphasis on SNORD115 increasing and SNORD116 decreasing (Laufer et al., 2013).

## Piwi-Interacting RNA

Piwi-Interacting RNAs (PiRNAs) are a diverse range of small RNAs that are highly enriched in the germline tissues. They interact with PIWI-class Argonaute proteins with sequence bias for only the first 5′ nucleotide to be a Uracil. This diverse population can be mapped back to distinct areas of the genome known as piRNA clusters, which contain highly enriched areas of fragmented dysfunctional transposable element (TE) sequences. These are thought to emanate from the memory of previous TE invasions, and can be utilized to protect against TEs (Toth et al., 2016). In addition, PIWI proteins function at the chromatin level by guiding DNA methylation and deposition of repressive histone marks to silence TE transcription (Le Thomas et al., 2013; Figure 1Dii).

The biogenesis of piRNAs gives rise to two different forms primary and secondary of 26–30 bps in length, stemming from single-stranded precursors (Yan et al., 2011; Mani and Juliano, 2013), which are best studied in Drosophila. Primary piRNAs biogenesis is poorly defined but precursors of around 200 bp stemming nearly entirely from piRNA clusters are cleaved – Zucchini (ZUC) is thought to do this – to enable loading onto a PIWI protein in association with other factors (Figure 1Di). This piRNA-PIWI complex interacts with TEs to prevent insertion through methylation or transcriptional repression, thereby affecting gene expression (Toth et al., 2016).

In Drosophila, secondary piRNAs are formed through a more defined “ping-pong” pathway, which utilizes the primary piRNAs formed from TE fragments present in piRNA clusters loaded onto Aubergine (AUB) to find complementary antisense TE transcripts (Figure 1D). Once found the complementary TE mRNA binds, and is cleaved ten nucleotides along from the 5′ end by AUB, which terminates its function. Additionally it creates a new 5′ end and piRNA precursor, which accompanied by AGO3 is processed into secondary piRNA. The secondary piRNA promotes the development of more cluster-derived piRNAs – it is representative of the sense TE strand – through complementary cluster transcripts to develop a greater repertoire against active TEs (Toth et al., 2016; Figure 1Di).

Originally piRNAs were solely thought to be present in germline cells, more recently they have been found in other areas of the body including blood (Yang et al., 2015), blood plasma (Freedman et al., 2016) and the brain (Roy et al., 2017) as well as interacting with diseases in the liver (Rizzo et al., 2016), cardiovascular system (Loche and Ozanne, 2016) and brain (Roy et al., 2017) demonstrating their roles are far-reaching. In neurodegenerative disease there have been recent studies on PD and AD.

Risk variants APOE (rs2075650) and RNU6-560P (rs10792835 + rs3851179) have been linked with AD through genome-wide association studies (GWAS). These risk variants were significantly correlated with nine (6 APOE and 3 RNU6-560P) different piRNAs, showing regulatory capabilities (Guo X. et al., 2017). PiRNA dysregulation may be integral to the development of AD through aberrant downstream signaling. The link to pathogenesis in AD was clarified in three AD dysregulated piRNAs (piR-38240, piR-34393, and piR-40666) after establishing complementary target genes (CYCS, KPNA6, and RAB11A) through inverse expression correlation (Roy et al., 2017). The target genes were known to regulate AD pathways through oxidative stress induced neurodegeneration, apoptosis and vesicular trafficking of Aβ. This demonstrates a regulatory role for piRNAs in preventing AD and so monitoring dysregulation could allow early diagnosis and implicate a treatment method.

There was a difference found in piRNA expression between PD- and control- patient derived cells. Patient tissue samples showed the same trend, with 70 different piRNAs overlapping between both (Table 3). Two distinct trends come from these piRNAs, up or down regulation (Schulze et al., 2018). In the down-regulated piRNA fraction, those that were short-interspersed nuclear elements (SINE) and long-interspersed nuclear elements (LINE) derived elements in cell lines and LINE in tissues, showed significant enrichment when compared to genome-wide expression (Schulze et al., 2018). This is indicative of an inability to silence SINE and LINE derived elements in PD-derived neurones, which could show a pathogenesis of PD disease.

## Transfer RNAs

Transfer RNAs (tRNAs) are the most abundant form of sncRNA, making up 4–10% of all cellular RNAs. Previously thought to be static contributors to gene expression, acting as an adaptor molecule in translation. Recently it has been found that small non-coding tRNAs have unique function that enable wider signaling and dynamic regulation of various functions (Gebetsberger and Polacek, 2013).

Mature tRNA is formed through transcription of precursor tRNA (pre-tRNA) using RNA polymerase III. Endonucleolytic ribonuclease P (RNase P) and ribonuclease Z cleave the transcribed pre-tRNA at the 5′ leader sequence and 3′ polyuracil (poly –U) tail, respectively, before tRNA nucleotidyl transferase adds a 3′CCA tail (Figure 1Ei). Many post-transcriptional modifications will occur during maturation and only tRNAs appropriately processed will leave the nucleus via nuclear receptor-mediated export process, with wrongly processed terminating. The mature tRNAs are between 73–90 nts in length and contain a clover-leaf shaped secondary structure, composing of a D-loop, an anticodon loop, a T-loop, a variable loop and an amino acid acceptor stem (Kirchner and Ignatova, 2015). The mature of pre-tRNA can be cleaved – into specific products unlike previously thought – into two main categories of cleaved tRNAs have been categorized; (1) tRNA-halves, (2) tRNA derived fragments.

tRNA halves are produced by cleavage of the anticodon loop giving rise to two halves; 30–35 nt 5′-tRNA halves and 40–50 nt 3′tRNA halves (Li and Hu, 2012; Figure 1Eii). A subtype of tRNA halves known as tRNA-derived stress-induced RNAs (tiRNAs) are by-products of stress. They induce cleavage by angiogenin (ANG) – a ribonuclease – of mature cytoplasmic tRNAs (Yamasaki et al., 2009).

tRNA derived fragments (tRFs) are produced from either pre-tRNAs or mature tRNAs (Figure 1Eii). Four main types have been established stemming from the fragment location on tRNAs: 5-tRFs, 3-tRFs, 1-tRFs, and 2 tRFs. 5-tRFs – located most abundantly in the nucleus – are generated from cleavage of the D-loop of tRNAs by Dicer, with adenine being present at the 3′ ends. Further subdivision classifies 5-tRFs isoforms into “a” (∼15 nts), “b” (∼22 nts) and “c” (∼30 nts) (Kumar et al., 2015; Lee et al., 2009). 3-tRFs result from cleavage by Dicer, ANG or another member of the Ribonuclease A superfamily of the T-loop, containing a CCA tail sequence (18–22 nts) (Lee et al., 2009; Maraia and Lamichhane, 2011; Kumar et al., 2015). 1-tRFs are formed by the cleavage of the 3′-trailer fragment of pre-tRNAs by either RNaseZ or ELAC2, this usually commences after the 3′-ends of mature tRNA and contains a poly-U 3′-end (Lee et al., 2009; Liao et al., 2010). 2-tRFs, less known about but may be formed from the anticodon loop (Goodarzi et al., 2015).

Numerous neurodegenerative disorders are associated with tRFs. ANG mutants show reduced ribonuclease (RNase) activity and were first implicated in the pathogenesis of amyotrophic lateral sclerosis (ALS) (Greenway et al., 2006). Latterly, a subset of the ALS-associated ANG mutants were observed in Parkinson’s disease (PD) patients (van Es et al., 2011). Recombinant ANG can improve life span and motor function in an ALS [SOD1 (G93A)] mouse model, demonstrating that tRFs may have an important role in motor neuron survival (Kieran et al., 2008) (Table 3).

The link between ANG-induced tiRNAs, cellular stress and neurodevelopment disorders was strengthened with the finding of NSun2 (Blanco et al., 2014). Mutations in the cytosine-5 RNA methyltransferase NSun2 have been shown to cause intellectual disability and a Dubowitz-like syndrome in humans (Abbasi-Moheb et al., 2012; Martinez et al., 2012). NSun2 methylates two different cytosine residues of tRNA. Without NSun2, cytosine-5 RNAs are not methylated, which increases the stress-induced ANG-mediated endonucleolytic cleavage of tRNAs and so 5′-tiRNAs accumulate. Accumulation of these factors leads to cell death in hippocampal and striatal neurons because of translational repression leading to cellular stress. Subsequently, NSun2 knockout mice show reduced neuronal size and impaired formation of synapses, which could explain the impairment of NSun2 gene mutation patients (Blanco et al., 2014).

A mutation in CLP1 gene (R140A) – a RNA kinase involved in tRNA splicing – is present in pontocerebellar hypoplasia (PCH) patients, a heterogeneous group of inherited neurodegenerative disorders characterized by the loss of motor neurons, muscle paralysis, impaired development of various parts of the brain and differential tRNA splicing (Karaca et al., 2014; Schaffer et al., 2014). The role of CLP1 in RNA splicing means the mutant gene has reduced kinase activity and affinity to the tRNA endonuclease complex (TSEN), impairing pre-tRNA cleavage and elevating unspliced pre-tRNAs in patient derived neurons (Schaffer et al., 2014). TSEN cuts the transcript at 3′ intron-extron junctions, so the absence of CLP1 means 5′-unphosphorylated tRF cannot interact with the pre-tRNA^tyr^ 3′-exon and subsequent splicing steps are interrupted (Cassandrini et al., 2010).

N^6^-threonyl-carbamoyl-adenosine (t^6^A) is a complex modification of adenosine involved in cytoplasmic tRNA modification. It is located next to the anticodon loop of many tRNAs that decode ANN codons, at position 37 (t^6^A37). Recently, a biosynthetic defect in the t^6^A molecule resulting from a mutation in the kinase-associated endopeptidase (KAE1) gene, which is part of the kinase, endopeptidase and other proteins of small size (KEOPS) complex was found in two phenotypically neurodegenerative patients, implicating tRNA modification in neuronal maintenance (Edvardson et al., 2017).

Although, tRNA-derived small non-coding RNAs, have already demonstrated a role in cancer progression (Sun et al., 2018), their role as biomarkers in NDs has not been fully investigated yet.

However, animal studies showed 13 dysregulated tRFs in brain samples of SAMP8 mouse model for AD. In particular, four were upregulated (AS-tDR-011775, AS-tDR-011438, AS-tDR-006835 and AStDR-005058) and 9 down regulated (AS-tDR-013428, AS-tDR-011389, AS-tDR-009392, AS-tDR012690, AS-tDR-010654, AS-tDR-008616, AS-tDR-010789, AS-tDR-011670, and AS-tDR-007919), demonstrating their potential involvement of tRFs in early detection of AD.

## Conclusion

The key problem with the ND field is the lack of understanding in the events preceding the development of protein-based markers – such as Tau – currently used to diagnose NDs. By this stage, the diseases become more difficult to treat.

SncRNAs play an important regulatory role in the maintenance of the homeostatic brain. Therefore, changes in their concentration levels can be indicative of mechanistic changes that could precede protein-based markers. One single sncRNA biomarker is unlikely to differentiate between diseases. However, a combination of sncRNA biomarkers could be illustrative of the mechanistic development of NDs to enable early diagnosis, enhanced disease monitoring as well as defining subtle differences between NDs. Consequently, novel treatment methods directly related to their mechanistic underpinning of specific NDs, and potentially other brain related pathologies can be envisaged.

Novel, less-well studied sncRNAs could be integral to understanding the overall disease progression. So new methodologies may be necessary to quantify these changes and allow for future biomarker development.

## Author Contributions

CW drafted the manuscript. AB and VDP critically revised the manuscript.

## Conflict of Interest Statement

The authors declare that the research was conducted in the absence of any commercial or financial relationships that could be construed as a potential conflict of interest.
